# Electronic Structure of Mg-, Si-, and Zn-Doped SnO_2_ Nanowires: Predictions from First Principles

**DOI:** 10.3390/ma17102193

**Published:** 2024-05-07

**Authors:** Alexander Platonenko, Sergei Piskunov, Thomas C.-K. Yang, Jurga Juodkazyte, Inta Isakoviča, Anatoli I. Popov, Diana Junisbekova, Zein Baimukhanov, Alma Dauletbekova

**Affiliations:** 1 Institute of Solid State Physics, University of Latvia, 8 Kengaraga Str., LV-1063 Riga, Latvia; a.platonenko@cfi.lu.lv (A.P.); intai@cfi.lu.lv (I.I.); popov@cfi.lu.lv (A.I.P.); 2 Department of Chemical Engineering and Biotechnology, National Taipei University of Technology, 1 Zhongxiao E. Rd. Sec. 3, Daan District, Taipei City 106, Taiwan; ckyang@mail.ntut.edu.tw; 3 Centre for Physical Sciences and Technology, Sauletekio Av. 3, LT-10257 Vilnius, Lithuania; jurga.juodkazyte@ftmc.lt; 4 Department of Technical Physics, L.N. Gumilyov Eurasian National University, Satpayev Str. 2, 010008 Astana, Kazakhstan; diana911115@gmail.com (D.J.); zeinb77@mail.ru (Z.B.); ak.dauletbekova@gmail.com (A.D.)

**Keywords:** SnO_2_, doped nanowires, density functional theory, ab initio calculation, electronic structure

## Abstract

We investigated the electronic structure of Mg-, Si-, and Zn-doped four-faceted [001]- and [110]-oriented SnO_2_ nanowires using first-principles calculations based on the linear combination of atomic orbitals (LCAO) method. This approach, employing atomic-centered Gaussian-type functions as a basis set, was combined with hybrid density functional theory (DFT). Our results show qualitative agreement in predicting the formation of stable point defects due to atom substitutions on the surface of the SnO_2_ nanowire. Doping induces substantial atomic relaxation in the nanowires, changes in the covalency of the dopant–oxygen bond, and additional charge redistribution between the dopant and nanowire. Furthermore, our calculations reveal a narrowing of the band gap resulting from the emergence of midgap states induced by the incorporated defects. This study provides insights into the altered electronic properties caused by Mg, Si, and Zn doping, contributing to the further design of SnO_2_ nanowires for advanced electronic, optoelectronic, photovoltaic, and photocatalytic applications.

## 1. Introduction

Tin dioxide (SnO_2_) is an n-type oxide semiconductor, boasting a myriad of advantageous physical and chemical properties that position it prominently in various technological applications [1]. It is characterized by a wide direct bandgap ($E_{g}=3.6$ eV at 300 K), an exceptional thermal and chemical stability, and environmental friendliness, aligning with the increasing demand for green and sustainable materials. Moreover, the cost-effectiveness of SnO_2_ amplifies its appeal for industrial applications. Just like other metal oxides such as ZnO, In_2_O_3_, and TiO_2_, SnO_2_ carves its niche in applications that span from being a transparent conductor to its role in oxidation catalysts, photocatalysts, photoluminescence, and solid-state gas-sensing materials [2,3,4,5,6,7,8,9,10,11,12,13,14,15,16,17,18,19,20,21]. These diverse applications are largely attributable to the unique surface properties of SnO_2_, which have become a focal point of extensive research and innovation.

In the past decade, the surge in interest towards SnO_2_ has been notably fueled by advancements in the synthesis of self-organized SnO_2_ nanostructures, especially nanowires (NWs), that exhibit large surface areas and enhanced reactivity [22,23,24,25]. These nanostructures not only inherit the intrinsic properties of SnO_2_ but are also endowed with additional features emerging from their nanoscale dimensions and morphological uniqueness. One of the most captivating facets of SnO_2_ NWs is the flexibility they offer in tuning the physical properties through controlled morphological modifications. This adaptability facilitates the engineering of nanomaterial-based devices tailored to address specific technological challenges and requirements, marking a significant stride towards the realization of customized solutions in electronics, optics, and sensing applications. The continuous evolution in synthesis techniques is paving the way for more predictable and reproducible modifications, augmenting the scope of applications and performance of SnO_2_-based devices.

The intrinsic properties of SnO_2_ nanomaterials are inherently influenced by factors such as morphology, the presence of impurities, and size effects intrinsic to the nanostructure. Doping emerges as a potent strategy to adeptly maneuver these properties, tailoring them for specialized applications [26]. A spectrum of doping elements has been explored, each imparting distinct characteristics to the SnO_2_-based materials and extending their applicability in the realms of optoelectronics and sensing [27]. N-type doping, exemplified by the incorporation of elements like F and Sb, has been demonstrated to enhance both the optical and electrical attributes of wide-bandgap SnO_2_ [28,29]. In this study, we explore the implications of Si doping, motivated by its cost-effectiveness and the potential for natural substitutional or interstitial defect incorporation during SnO_2_ nanowire synthesis utilizing silicon nanoporous templates [30]. Optical transmittance is a critical parameter, and Si-doped SnO_2_ thin films are postulated to exhibit an enhanced performance, attributed to the wide bandgap nature of SiO_2_ ($E_{g}=8.9$ eV) [31]. On the other hand, acceptor dopants such as zinc are garnering attention for their ability to increase hole concentration without significantly altering the lattice constants, thanks to the comparable ionic radii of Zn^2+^ and Sn^4+^ ions of 0.74 and 0.69 Å, respectively [32]. Despite these promising prospects, the literature on Zn-doped SnO_2_ nanomaterials remains scant [33], prompting a deeper exploration into their potential and characteristics. Alkaline earth metal-doped SnO_2_, particularly with elements like Mg, has been relatively underexplored. Mg introduces a distinct dynamic, given its spin-polarized 2p states in the doped state, contributing to an uncommon form of conductivity in this transparent conducting oxide [34]. The nuanced conductivity profile of SnO_2_ is attributed to the intricate interplay between tin interstitial and oxygen vacancies, compounded by the nanostructure morphology. This has positioned SnO_2_ as a frontrunner in gas-sensing materials [35]. The debate regarding the viability of p-type doping through the introduction of acceptors on the cationic site is ongoing, with divergent perspectives emerging in the recent literature [36,37]. Zhang et al. highlighted the low formation energy (0.48 eV) for Mg-doped SnO_2_, indicating the ease of its experimental synthesis and augmenting its feasibility for diverse applications [34].

In this work, we endeavor to provide an in-depth analysis of the electronic structure of SnO_2_ nanowires, with an emphasis on the effects instigated by substitutional doping with magnesium (Mg), silicon (Si), and zinc (Zn) atoms. Our approach incorporates first-principles calculations executed within the purview of hybrid density functional theory (DFT), offering precise and comprehensive insights into the electronic properties engendered by defect-induced effects in these doped nanowires.

Our focus on Mg, Si, and Zn as dopants is motivated by their distinct attributes and the differential impacts they exert on the electronic and optical characteristics of SnO_2_ nanowires. Mg, for instance, with its electronic configuration and ionization potential, introduces electronic states that can be instrumental in tuning the electronic and optical properties of tin oxide nanowires. Si, a common element in semiconductor technology, is envisaged to impact the charge carrier mobility and enhance the structural stability of SnO_2_ nanomaterials. Zn, with its ability to modulate the bandgap while maintaining lattice congruence, offers prospects for optimizing the optoelectronic properties of these nanostructures.

An understanding of the electronic properties is instrumental for unveiling the potential of SnO_2_ nanowires in a number of applications, including optoelectronic devices, gas sensors, and photocatalysts. By unraveling the correlations between the type and concentration of dopants and the ensuing modifications in the electronic structure, we aim to give a suggestion for the rational design and potential synthesis of SnO_2_ nanowires with tailored properties. We expect that our study will evaluate the stability, formation energy, and potential morphological changes induced by doping, and thus examine the link between atomic-scale interactions and macroscopic properties, enabling the precise customization of SnO_2_ nanowires for targeted applications. Our calculations aim to substantially enrich the existing knowledge base, driving the creation of advanced devices with an improved performance and efficiency and a broader range of functionalities.

This paper begins with an explanation of the method employed, specifically detailing our choice of a DFT-based approach. We then present our results, focusing on calculations for two distinct nanowire configurations and the subsequent electronic and energetic properties derived. The discussion and interpretation of obtained results, highlighting the impacts of doping on the SnO_2_ nanowires, are presented in the discussion. A short summary of our theoretical study is given in the conclusions.

## 2. Computational Details

First-principles calculations on doped SnO_2_ NWs were conducted utilizing the localized Gaussian-type function (GTF) formalism. This method expands the crystalline orbitals of the *N*-electron system into linear combinations of a set of *m* Bloch functions, which are constructed from atom-centered GTFs. This is achieved using the linear combination of atomic orbitals (LCAO) approach within the density functional theory (DFT) framework. Previously, we have effectively employed this computational approach in first-principles simulations of other metal oxides and more complex materials [38,39,40,41,42,43]. The first-principles DFT-LCAO method, as implemented in the CRYSTAL code [44,45], is able to evaluate both 2D slabs (surfaces) and 1D nanowires without imposing artificial 3D periodicity. Our calculations on all SnO_2_ materials under study incorporated the hybrid Hartree–Fock/Kohn–Sham (HF/KS) exchange-correlation functional PBE0 [46] mixing the exact HF non-local exchange and KS exchange operator within the generalized gradient approximation (GGA). In our study, PBE0 was employed to enhance the reliability of our band structure calculations. All-valence double–ζ GTF basis sets (BSs) were applied for oxygen, magnesium, silicon, and zinc atomic species [47], while the tin atom was calculated using a triple–ζ BS, adopting an effective core pseudopotential (ECP) [48]. Spin-polarized calculations were performed for Zn- and Mg-doped SnO_2_ nanowires. To provide the balanced summation over the direct and reciprocal lattices, reciprocal space integration was performed by sampling the Brillouin zone (BZ) with the 8×8×8 Pack–Monkhorst k-mesh [49] that resulted in 75 evenly distributed k-points in the irreducible BZ (IBZ) of bulk SnO_2_, with the 8×8×1 k-mesh or 21 k-points in the IBZ of the most stable SnO_2_ (001) and (110) surfaces calculated through the 2D slab model [44,45], and with the 4×1×1 k-mesh or 3 k-points in the IBZ of the both doped and pristine 1D SnO_2_ nanowires. The threshold parameters of CRYSTAL code (ITOLn) for assessment of bielectronic integrals (overlap and penetration tolerances for Coulomb integrals, ITOL1 and ITOL2, overlap tolerance for exchange integrals ITOL3, and pseudo-overlap tolerances for exchange integral series, ITOL4 and ITOL5) [44,45] were set to 7, 7, 7, 7, and 14, respectively. (If the overlap between the two atomic orbitals is less than $10^{-ITOLn}$, the corresponding integral is neglected.) Increasing the k-mesh and threshold parameters further led to significantly more computational expense with only a marginal improvement in the total energy accuracy (≤$10^{-7}$ a.u.). Convergence in the calculations was achieved when the variation in total energy, obtained through the self-consistent field (SCF) procedure, was less than $10^{-7}$ a.u. between two successive cycles. Every SnO_2_ structure calculation underwent total geometry optimization, with a convergence criterion set to a total energy difference of less than $10^{-6}$ a.u. across two successive SCF cycles. Effective charges on atoms and net bond populations were analyzed using the Mulliken population analysis [44], offering insights into the charge distribution and bonding characteristics within the doped SnO_2_ NWs.

Our study focuses on the rutile polymorph of SnO_2_, noted for its stability [50]. Characterized by a tetragonal lattice and space group $P4_{2}/mnm$, the rutile structure of tin oxide consists of two SnO_2_ formula units within each primitive unit cell. The structural parameters of the SnO_2_ rutile bulk calculated in this study ($a_{0}=4.731$ Å, $c_{0}=3.193$ Å and u=0.307) are in good qualitative and quantitative agreement with those measured experimentally ($a_{0}=4.737$ Å, $c_{0}=3.185$ Å and u=0.307 [51]), thus indicating reliability of the optimization procedure and chosen theoretical approach. Calculation by means of PBE0 optical band gap energy ($E_{g}=3.91$ eV, direct Γ−Γ gap) just slightly overestimates the band gap measured experimentally (3.6 eV [1]). Sn–O bond lengths were defined inside the first (I) and second (II) coordination spheres consisting of four and two oxygen atoms around each tin atom. The calculated Sn–$O^{I}$ and Sn–$O^{\mathrm{II}}$ bond lengths are 2.053 Å and 2.055 Å, respectively. The calculated Sn–$O^{I}$ and Sn–$O^{\mathrm{II}}$ bond populations are 210 me and 276 me, respectively.

**Figure 1:**
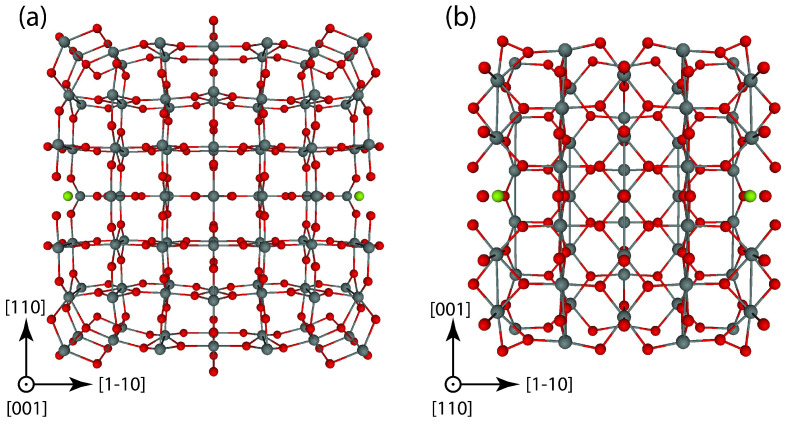
Schematic view of optimized periodically repeated four-faceted SnO_2_ NW unit cell (UC) of rutile morphology: (**a**) [001]-oriented 1D NW with 98 formula units per UC and (**b**) [110]-oriented 1D NW with 55 formula units per UC. Tin atoms are depicted as grey balls, while red balls stand for oxygen atoms. Dopant atoms substituted for host Sn are shown as green balls.

Nanowires, akin to infinite prisms with varying diameters, derive their stability from the stability of their lateral facets, consistent with the principles of Wulff’s rules [52]. This concept suggests that for rutile SnO_2_, the cross-sections of the nanowires represent configurations with the fewest dangling bonds per facet surface unit cell. Evarestov and Zhukovskii have provided a detailed description of the formation mechanism for rutile-type 1D nanowires, taking into account the stability of nanowire’s facets originating from 2D slabs [53]. According to the authors’ prescriptions, the stability of the rutile-type nanowire is governed by the stability of its lateral facets. In agreement with recent experimental studies [54,55], our calculations predict that both SnO_2_ (001) and (110) surfaces will have the lowest surface energy with respect to the bulk phase, i.e., 0.154 and 0.097 eV/Å^2^, respectively. Therefore, in our study, we have modeled [001]-oriented SnO_2_ NWs terminated by {110} and {1–10} facets consisting of 98 formula units per unit cell (Figure 1a) and [110]-oriented NWs terminated by alternating {1–10} and {001} facets consisting of 55 formula units per unit cell (Figure 1b). These are the only SnO_2_ NW morphologies considered in this study. The formation energy per formula unit with respect to rutile phase of SnO_2_ bulk calculated for [001]-oriented NWs (0.52 eV) is lower than formation energy calculated for [110]-oriented SnO_2_ NWs (1.11 eV), mainly due to size effects, as the periodically repeated 1D UC of [110]-oriented NW is smaller.

In order to keep the symmetry, Mg, Si, or Zn atoms were substituted for two host Sn atoms at the NW’s surface from both sides, as it is shown in Figure 1. For all equilibrium geometries of doped SnO_2_ NWs, calculations of phonon modes were performed to avoid the presence of imaginary frequencies. For [001]-oriented NWs, the dopant concentration is estimated as 2.0%, while for [110]-oriented NWs, the dopant concentration is 3.6%. The formation energy of a substitutional dopant in SnO_2_ NWs was calculated using the following relation: $E_{\mathrm{form}}=E_{\mathrm{NW}}^{\mathrm{doped}}+E_{\mathrm{Sn}}-E_{\mathrm{dopant}}-E_{\mathrm{NW}}^{\mathrm{pristine}}$, where $E_{\mathrm{NW}}^{\mathrm{doped}}$ is the total energy calculated for [001]- or [110]-oriented SnO_2_ NWs containing Mg, Si, or Zn dopant atoms (substitutional point defect), $E_{\mathrm{Sn}}$ is the total energy calculated for the most stable β-Sn crystalline solid, $E_{\mathrm{dopant}}$ is the total energy calculated for the most stable polymorph of dopant solid, and $E_{\mathrm{NW}}^{\mathrm{pristine}}$ is the total energy calculated for the pristine [001]- or [110]-oriented SnO_2_ NWs. By considering the energetics of the individual components (pristine nanowire, dopant atom, and free-standing host atom), the formation energy can be accurately determined, providing valuable insights into the stability and feasibility of the doped SnO_2_ nanowire structures.

## 3. Results and Discussion

**Figure 2:**
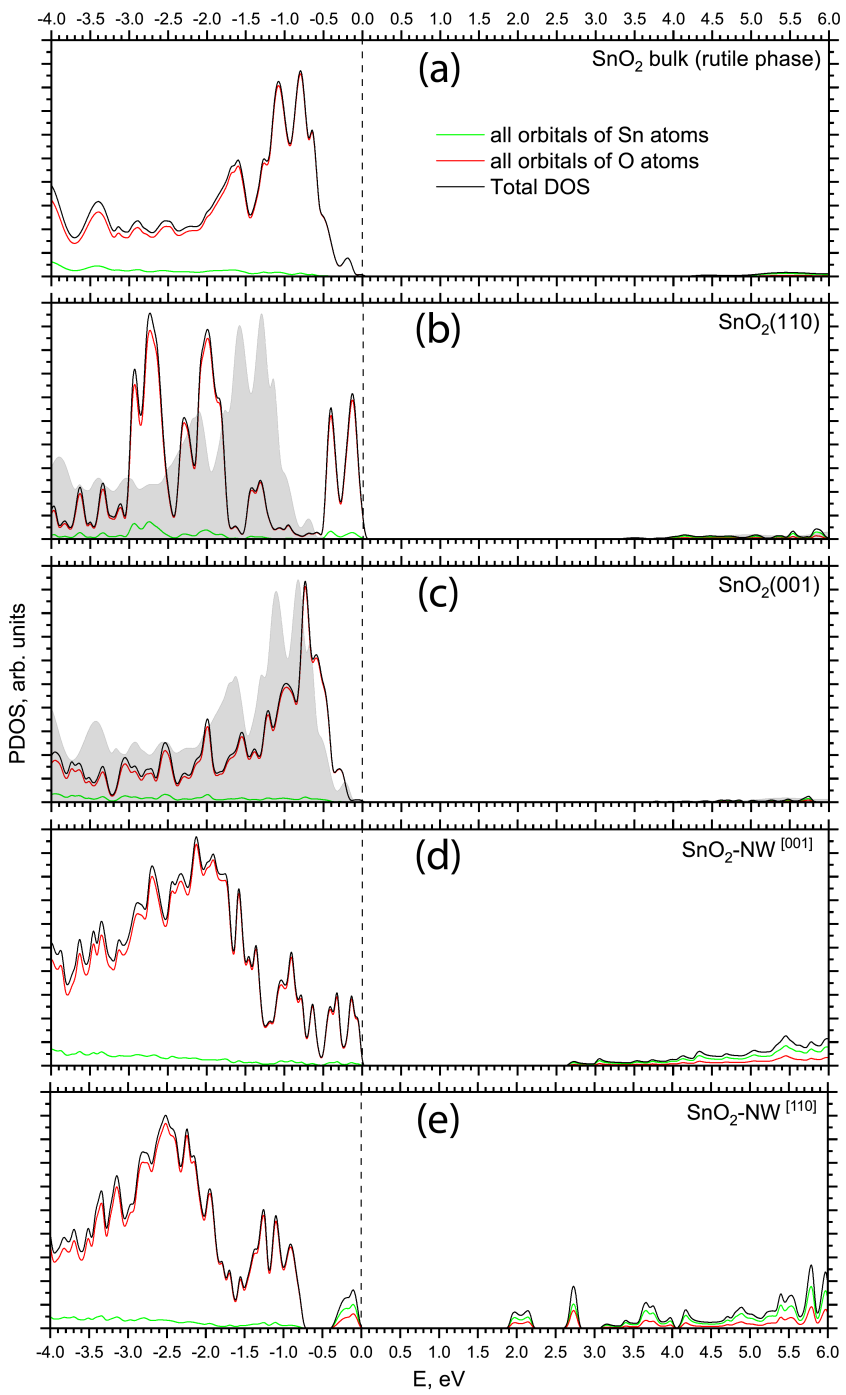
Total and projected density of states (PDOS) of SnO_2_ materials as calculated by means of PBE0 exchange-correlation functional within DFT: (**a**) rutile SnO_2_ bulk phase, (**b**) SnO_2_ (110) surface calculated through the slab model, (**c**) SnO_2_ (001) surface calculated through the slab model, (**d**) pristine [001]-oriented SnO_2_ NW (see Figure 1a), (**e**) pristine [110]-oriented SnO_2_ NW (see Figure 1b). The energy scale is referenced relative to the valence band maximum. DOSes are projected onto all orbitals of all corresponding atoms. To illustrate the influence of surface states on the slab’s electronic structure, the background in (**b**,**c**) displays the total DOS of the SnO_2_ rutile bulk with its highest occupied orbital energy aligned with the highest occupied orbital energy of atoms from the central atomic layer of the corresponding slab.

Figure 2 compares the total and projected densities of states (PDOSs) calculated in this study for the pristine SnO_2_ bulk of the rutile phase (Figure 2a), stoichiometric SnO_2_ (110) and (001) surfaces (Figure 2b,c), and [001] and [110]-oriented pristine SnO_2_ nanowires (Figure 2d,e). The energy scale on all PDOS graphs is shown with respect to the top of the valence band. The top of the valence band of the crystalline SnO_2_ is formed solely by O 2p states. The conduction band bottom is dominated by Sn 5s orbitals with a negligible contribution from O 2p states. The upper part of the conduction band of crystalline SnO_2_ is dominated by Sn 5p states. The valence and conduction bands of bulk SnO_2_ are separated by a direct Γ−Γ band gap of 3.91 eV (Table 1).

Stoichiometric surfaces arise from cleaving the bulk crystal along specific crystallographic planes, ensuring an equal number of severed bonds between tin and oxygen atoms, thereby preserving their respective oxidation states. Pristine crystalline SnO_2_ displays a variety of facets, with the (110) and (001) surfaces being the most predominant energetically. On the stoichiometric (110) surface, oxygen atoms are presented in two distinct coordinations: those in a threefold coordination within the plane, and those in a twofold coordination serving as bridging atoms. The top of the valence band in the PDOS calculated for the SnO_2_ (110) surface is predominantly formed by O 2p orbitals, with small contributions from Sn 5s states. The conduction band bottom consists of an admixture of Sn 5s and O 2p orbitals. The (110) surface states are formed by 2p electrons of the bridging surface oxygen atoms. An indirect band gap of 3.22 eV is predicted for the SnO_2_ (110) surface in this study.

The SnO_2_ (001) surface consists of two Sn–O_2_ atomic planes in periodically repeating layer units, in which the summed electric charge is neutral. The electronic structure of the SnO_2_ (001) surface is the same as for crystalline SnO_2_. A direct Γ−Γ band gap of 3.46 eV is calculated for the (001) surface.

In the rutile-structured tin oxide nanowire oriented along the [001] direction, as shown in Figure 1a, the translational axis is perpendicular to both the (110) and (1–10) surfaces that define the square cross-section. Therefore, the electronic structure calculated for the pristine [001]-oriented SnO_2_ NW is similar to that of the pristine (110) slab. An indirect band gap of 2.72 eV is predicted for the [001]-oriented SnO_2_ NW.

The periodic translation axis of the SnO_2_ nanowire oriented along the [110] direction is perpendicular to the surfaces of the slab, and the structural genesis of this orientation is found to be more intricate compared to the [001]-oriented nanowire. It features lateral facets including the (001), (00–1), (110), and (–110) planes, as illustrated in Figure 1b. Despite significant atomic relaxation, rutile-type SnO_2_ nanowires oriented along the [110] direction exhibit notably higher formation energies (with respect to the bulk phase) compared to those oriented along the [001] direction, with values of 1.11 eV versus 0.52 eV, respectively. The valence-band maximum of the pristine [110]-oriented NW is derived from the O 2p orbitals hybridized with Sn 5s states, forming a peak separated from the valence band by 0.31 eV. The conduction band minimum is located 3.12 eV above the top of the valence band and consists mainly of Sn 5s states. Geometry relaxation of the [110]-oriented NW results in a substantial atomic redistribution and formation of sharp nanowire edges (Figure 1b). This results in two shallow empty states at 0.25 and 0.79 eV below the bottom of the conduction band (Figure 2e). These peaks consist of an admixture of oxygen 2p and tin 5s electronic orbitals.

Calculation of the Sn–O bond lengths in crystalline SnO_2_ and on the (110) and (001) surfaces reveals a significant contraction of these bonds when transitioning from the bulk to the surface. In the bulk SnO_2_, the Sn–O bond length is 2.05 Å, while on the (110) and (001) surfaces, the bond lengths decrease to 1.96 Å and 1.93 Å, respectively, as detailed in Table 1. This reduction in bond length correlates with an enhancement in bond covalency, which is a result of considerable charge redistribution in the system. The bond population calculations indicate values of 432 and 556 millielectrons for the Sn–O bond on the pristine (110) and (001) surfaces, respectively, figures that are more than double the bond population found in the rutile bulk phase. Given that the facets of the nanowires are composed of (110) or (001) surfaces, it follows that the chemical characteristics of the Sn–O bonds on the surface of pristine SnO_2_ nanowires mirror those of their respective surfaces, as demonstrated in Table 1.

**Figure 3:**
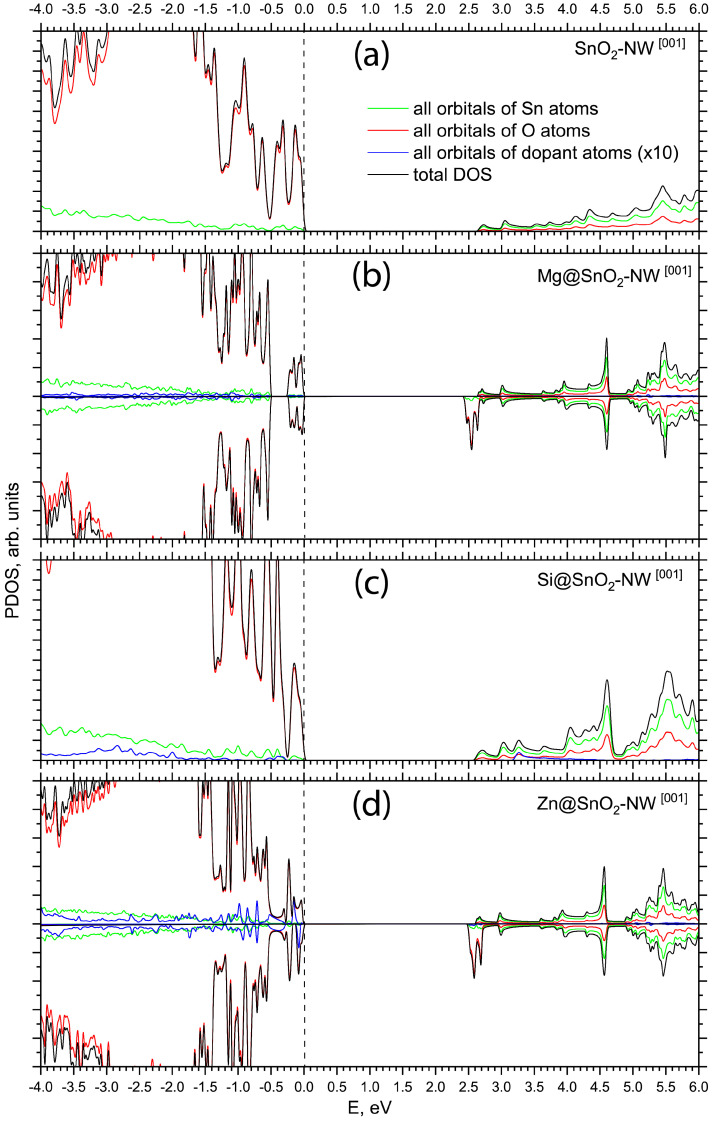
Total and projected density of states (PDOS) of pristine and doped [001]-oriented SnO_2_ NWs as calculated by means of PBE0 exchange-correlation functional within DFT: (**a**) pristine [001]-oriented SnO_2_ NW, (**b**) Mg-doped [001]-oriented SnO_2_ NW (upper panel stands for spin-up electrons, lower panel stands for spin-down electrons), (**c**) Si-doped [001]-oriented SnO_2_ NW, (**d**) Zn-doped [001]-oriented SnO_2_ NW (upper panel stands for spin-up electrons, lower panel stands for spin-down electrons). The energy scale is referenced relative to the valence band maximum. PDOS peaks associated with dopant atoms are magnified tenfold.

Figure 3 compares the total and projected densities of states (PDOSs) calculated in this study for pristine [001]-oriented SnO_2_ NWs (Figure 1a) and [001]-oriented SnO_2_ NWs with Mg, Si, or Zn substituted for the host Sn atom on the nanowire’s surfaces. Substituting Mg for Sn in the rutile structure of [001]-oriented SnO_2_ nanowires is predicted to notably affect the valence band, although the conduction band is largely unaltered when compared to the undoped [001]-oriented SnO_2_ nanowires (Figure 3b). The incorporation of the *s* and *p* orbitals from the Mg dopant leads to significant hybridization with the O 2p orbitals, which in turn distinctly reshapes the projected density of states (PDOS) near the valence band maximum. Such theoretical insights are crucial for understanding the interplay between the electronic structure and the anticipated electronic characteristics of the material. Despite these alterations in the electronic structure, the calculated indirect band gap for the Mg-doped [001]-oriented nanowire remains close to that of the pristine one, with a value of 2.47 eV for the doped nanowire as opposed to 2.72 eV for the undoped [001]-oriented SnO_2_ nanowire, as listed in Table 1. According to the Mulliken population analysis performed for both [001]- and [110]-oriented non-optimized Mg-doped SnO_2_ NWs, Mg donates three electrons (Mg^3+^) to the closest host oxygens, keeping the unit cell neutral. The optimized Mg–O bond length for the substitutional Mg dopant within the [001]-oriented SnO_2_ nanowire, as reported in Table 1, reveals an elongation to 2.00 Å compared to the Sn–O bond length of 1.97 Å found in the pristine nanowire. This increment in the Mg–O bond distance leads to a decrease in covalency and an increase in the effective negative charge on the adjacent oxygen atoms with respect to an undoped nanowire.

The projected density of states calculated for a [001]-oriented SnO_2_ nanowire with an isoelectronic Si substitutional dopant is presented in Figure 3c. Silicon is identified as an energetically favorable dopant, exhibiting a low formation energy of 0.72 eV, as listed in Table 1, which suggests its potential incorporation into SnO_2_ nanowires synthesized using silicon nanoporous templates through the track template method [56]. The top of the valence band of the Si-doped [001]-oriented SnO_2_ nanowire is primarily composed of O 2p orbitals, with a minor contribution from Sn 5s and Si 3s states. The bottom of the conduction band is predominantly characterized by Sn 5s with a small fraction of O 2p states. The calculated indirect band gap of the Si-doped [001]-oriented SnO_2_ NW (2.68 eV) is practically unchanged with respect to the band gap calculated for the pristine nanowire (2.72 eV).

The atomic mass is strongly correlated with the atomic number (nuclear charge) and thus influences both the bond length and strength of the bond. Atoms with a larger atomic number tend to form longer bonds due to their larger size. The bond length inversely affects the bond strength and covalency. Shorter bonds are generally stronger and more covalent. The smaller atomic mass of Si compared to Sn results in a shorter Si–O bond length of 1.73 Å, leading to a higher covalency, as indicated by the Si–O bond population of 510 millielectrons.

Substituting a Zn atom for Sn at the (110) facet of a [001]-oriented SnO_2_ nanowire induces states near the valence band maximum, as depicted in Figure 3d. These states are mainly an admixture of the host O 2p orbitals and Zn 3d levels, whereas the valence band itself predominantly comprises O 2p orbitals hybridized with Sn 5s and Zn 3d states. Our calculations confirm the involvement of both Zn 3d and 4s orbitals in the electronic structure of the doped nanowires, indicating that both orbitals play a role in the hybridization with the host’s O 2p orbitals. However, this specifically emphasizes the contribution of Zn 3d orbitals to the valence band maximum, suggesting that the Zn 3d orbitals have a more pronounced impact on the electronic properties of the doped nanowires than the Zn 4s orbitals. The calculated indirect band gap for the Zn-doped [001]-oriented nanowire remains at 2.53 eV, close to the value calculated for the pristine nanowire. The Zn–O bond length in the doped nanowire is slightly longer than the Sn–O bond in the undoped SnO_2_ NW, at 2.02 Å compared to 1.97 Å. A Mulliken population analysis performed for both [001]- and [110]-oriented non-optimized Zn-doped SnO_2_ NWs shows that Zn donates three electrons (Zn^3+^) to the closest host oxygens, keeping the unit cell neutral. The doping-induced charge redistribution yields a Zn–O bond that is less covalent, with a bond population of 212 millielectrons, as listed in Table 1. The formation energy for the Zn dopant is calculated to be 5.07 eV, indicating that Zn doping is energetically less favorable and likely an endothermic process.

**Figure 4:**
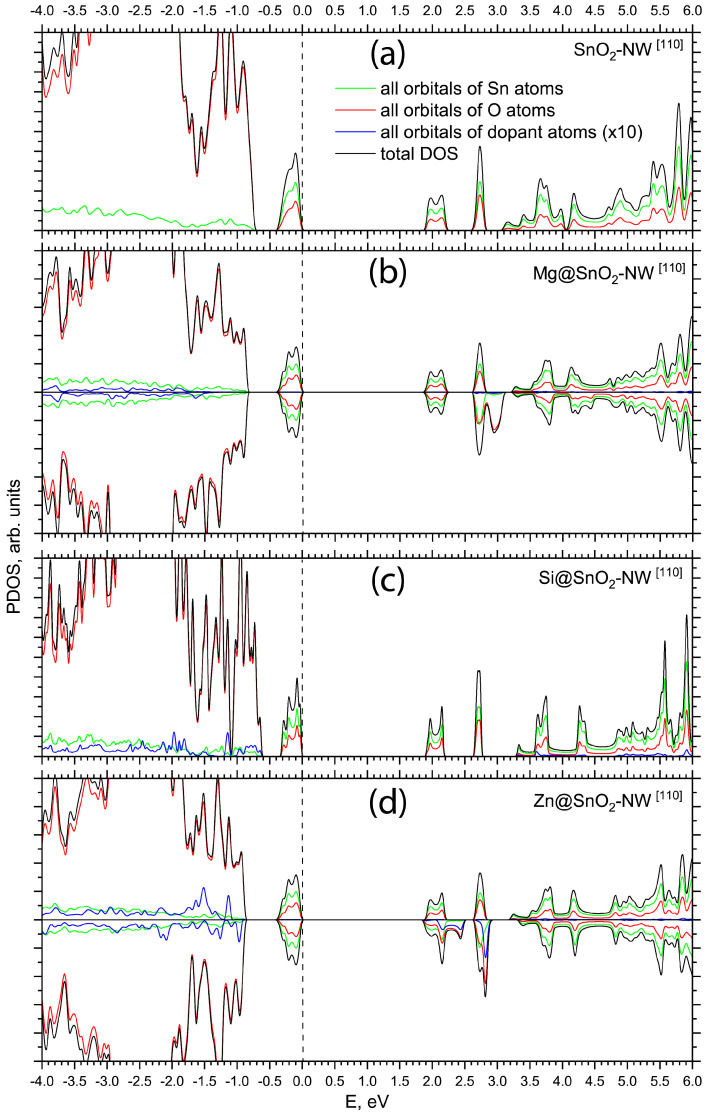
Total and projected density of states (PDOS) of pristine and doped [110]-oriented SnO_2_ NWs as calculated by means of PBE0 exchange-correlation functional within DFT: (**a**) pristine [110]-oriented SnO_2_ NW, (**b**) Mg-doped [110]-oriented SnO_2_ NW (upper panel stands for spin-up electrons, lower panel stands for spin-down electrons), (**c**) Si-doped [110]-oriented SnO_2_ NW, (**d**) Zn-doped [110]-oriented SnO_2_ NW (upper panel stands for spin-up electrons, lower panel stands for spin-down electrons). The energy scale is referenced relative to the valence band maximum. PDOS peaks associated with dopant atoms are magnified tenfold.

Figure 4 presents a comparative analysis of the total and projected densities of states for the pristine [110]-oriented SnO_2_ nanowire (NW) (Figure 1a) and [110]-oriented SnO_2_ NWs with Mg, Si, or Zn substitutions at the host Sn atom positions on the nanowire surfaces. Following doping, the [110]-oriented SnO_2_ NW largely retains the electronic structure of the pristine [110]-oriented nanowire, featuring two shallow empty states at 0.25 and 0.79 eV below the conduction band bottom (Figure 4a–d). These states are characterized by a mixture of oxygen 2p and tin 5s electronic orbitals. Alterations in the top of the valence band induced by doping the [110]-oriented NW closely resemble those observed in [001]-oriented SnO_2_ NWs. Specifically, Mg substitution for the host Sn atom in the [110]-oriented NW results in an indirect band gap of 3.26 eV, compared to the pristine [110]-oriented NW gap of 3.12 eV (Table 1), accompanied by the emergence of hybridized O 2p and Sn 5s states near the top of the valence band (Figure 4b). The optimized Mg–O bond length for the substitutional Mg dopant in the [110]-oriented SnO_2_ nanowire, as listed in Table 1, exhibits elongation to 2.04 Å, contrasting with the Sn–O bond length of 1.97 Å in the pristine nanowire. This elongation leads to a significant reduction in covalency, with a bond population of 142 millielectrons, compared to the undoped nanowire. The defect formation energy calculated for Mg-doped [110]-oriented nanowire is approximately 0.3 eV higher than that for Mg dopant in the [001]-oriented SnO_2_ NW.

The impact of an isoelectronic Si substitutional dopant on the electronic structure of [110]-oriented SnO_2_ nanowires closely mirrors the changes predicted for [001]-oriented SnO_2_ nanowires (Figure 4c). In the Si-doped [110]-oriented SnO_2_ nanowire, the top of the valence band is predominantly composed of O 2p orbitals, with a minor contribution from Sn 5s and Si 3s states. The calculated indirect band gap of the Si-doped [110]-oriented SnO_2_ NW slightly increases to 3.31 eV compared to the pristine nanowire’s band gap of 3.12 eV. Due to the smaller atomic mass of Si compared to Sn, the Si–O bond length in the doped nanowire is shortened to 1.70 Å, resulting in a higher covalency, as indicated by the Si–O bond population of 714 millielectrons. A low defect formation energy of 0.32 eV is predicted for Si doping in the [110]-oriented SnO_2_ NW.

Introducing a Zn atom as a substitutional dopant for Sn at the (1–10) facet of a [110]-oriented SnO_2_ nanowire induces occupied states located close the valence band maximum similar to the Mg dopant, as illustrated in Figure 4d. This state is predominantly composed of Zn 3d electronic orbitals. The calculated indirect band gap for the Zn-doped [110]-oriented nanowire is 3.32 eV. In the doped nanowire, the Zn–O bond length is slightly shorter than the Sn–O bond in the undoped SnO_2_ NW, measuring 1.93 Å compared to 1.97 Å. Doping-induced charge redistribution results in a Zn–O bond that is less covalent, with a bond population of 224 millielectrons, as listed in Table 1. The formation energy for the Zn dopant is calculated to be 6.09 eV.

Calculated PDOSs for Mg- and Zn-doped nanowires (Figure 3b,d and Figure 4b,d) show splitting between the spin-up and spin-down subbands, which implies doping can result in magnetism in the SnO_2_ nanowire. The magnetic moments are mainly contributed by the Mg 2p and Zn 3d orbitals with an admixture from the orbitals of the nearest neighboring O and Sn atoms.

While we note that the formation energy of the dopants is an important consideration, in our study, we pay attention to dopants with formation energies above 2 eV. The formation energy is not the sole determining factor for the experimental feasibility of doping. Other factors, such as the synthesis method, temperature, and pressure conditions, can significantly influence the incorporation of dopants into the host material [57]. Dopants with relatively high formation energies also can be incorporated into various materials, leading to desirable modifications in their electronic, optical, and catalytic properties [58].

In summary, we emphasize that the electronic properties of rutile-based SnO_2_ nanowires exhibit both size and shape dependence. Our first-principles calculations, employing hybrid DFT methods, affirm that the stability of nanowires is predominantly determined by the stability of their lateral facets. Consequently, [001]-oriented SnO_2_ nanowires terminated by (110) and (1–10) facets are energetically more favorable than [110]-oriented nanowires terminated by alternating (1–10) and (001) facets. The bandgap of SnO_2_ nanowires is strongly influenced by their crystallographic orientation, the stability of their surface facets, the type and concentration of dopants, and the resulting atomic-scale structural changes induced by doping. Doping SnO_2_ nanowires induces electronic changes at the bottom of the conduction band, resulting in lowered band edge positions. Thus, the incorporation of alkaline earth metal doping into an electron-rich semiconductor holds promise for achieving novel photoluminescence and photocatalytic activities in future applications. These effects can be observed through optical and photoelectron spectroscopy methods, as well as by measuring the electrical properties of the SnO_2_ nanowires. The presence of midgap levels within the optical bandgap of defective nanowires makes them appealing for bandgap engineering, particularly in photocatalytic applications. The results reported in this work elucidate the tuning effects of 3d-metal doping on the optical, dielectric, and magnetic properties of SnO_2_ nanowires. This approach is extendable to other wide-bandgap semiconductors, offering opportunities for enhancing the properties of optoelectronic and spintronic devices. Furthermore, the photocatalytic activities of both doped and undoped SnO_2_ nanowires can be evaluated by studying the photodegradation of organic pollutants and photocatalytic water splitting. The smaller size of the nanowires may result in improved light-harvesting capabilities, and the anticipated light absorption in the visible range at optimum dopant concentrations opens new prospects for degrading organic pollutants and photocatalytic hydrogen production under solar irradiation.

## 4. Conclusions

In this study, extensive first-principles calculations were conducted to investigate the electronic structure of [001]- and [110]-oriented SnO_2_ nanowires (NWs) incorporating substitutional impurity atoms, namely Mg, Si, and Zn. Our calculations employed the linear combination of atomic orbitals (LCAO) with atomic-centered Gaussian-type functions as the basis set. The theoretical predictions, in good qualitative agreement, indicate low formation energies, implying the relative stability of point defects resulting from atom substitutions at the surfaces of SnO_2_ NWs. Furthermore, the formation energies of individual substitutional point defects on relaxed SnO_2_ NWs increased proportionally with the atomic mass of the substitutional defect. The low formation energy predicted for the isoelectronic substitutional Si atom suggests straightforward incorporation of the dopant during synthesis. Analysis of equilibrium distances between substitutional defects and the host oxygen atoms reveals variations in bond lengths compared to the Sn–O bond length in pristine SnO_2_ NWs. This relaxation is accompanied by slight changes in covalency and additional charge redistribution between the defect and the nanowire. The calculated density of states indicates the emergence of midgap states within the band gap of SnO_2_ NWs, resulting in gap narrowing. Based on our quantum chemical calculations, we conclude that dopants significantly influence the band structure of SnO_2_ NWs. Mg doping introduces states near the valence band maximum, narrowing the band gap and resulting in elongated Mg-O bond lengths, a decreased covalency, and an increased negative charge on adjacent oxygen atoms. It has a relatively low formation energy, suggesting easier experimental synthesis. Si doping is predicted to be energetically favorable, with a negative formation energy. Si doping maintains the wide-bandgap nature of SnO_2_ nanowires, making it an attractive option for applications requiring wide-bandgap materials. Zn doping introduces occupied states near the valence band maximum, narrowing the band gap. Zn doping results in slightly shorter Zn-O bond lengths but less covalent bonds. The formation energy for Zn doping is relatively high, indicating challenges in incorporation compared to Mg and Si. Based on these predictions, Si doping appears to be the most promising candidate for maintaining the wide-bandgap nature of SnO_2_ nanowires, which is crucial for applications in optoelectronics, photocatalysis, and gas sensing. Si doping’s energetically favorable process and its ability to maintain the wide-bandgap nature of SnO_2_ make it an attractive option for enhancing the electronic and optical characteristics of SnO_2_ nanowires. This effect is crucial to consider when designing nanoelectronic and photocatalytic devices based on these nanowires. We propose that the observed effects can be experimentally validated using optical and photoelectron spectroscopy methods, as well as by assessing the (photo)electrochemical properties of SnO_2_ NWs.

## Figures and Tables

**Table 1:** Energy of substitutional defect (dopant) formation (*E*_from_ in eV), Sn–O or dopant–O bond length (*l*_Sn/dop−O_ in Å, shortest atomic bond), Sn–O or dopant–O bond population (*P*_Sn/dop−O_ in me (milli e), shortest atomic bond), Mulliken effective charges of Sn or dopant and O atoms (*Q*_Sn/dop/O_), and band gap ($E_{g}$ in eV) as calculated in the present study.

Material	$E_{\mathrm{form}}$	*l*_Sn/dop−O_	*P*_Sn/dop−O_	*Q*_Sn/dop_	$Q_{O}$	$E_{g}$
Mg@SnO_2_-NW^[001]^	2.03	2.000	166	1.47	–1.06	2.47
Si@SnO_2_-NW^[001]^	0.72	1.730	510	1.67	–0.95	2.68
Zn@SnO_2_-NW^[001]^	5.07	2.020	212	1.40	–0.88	2.53
Pristine SnO_2_-NW^[001]^		1.967	430	1.89	–0.91	2.72
Mg@SnO_2_-NW^[110]^	2.34	2.038	142	1.47	–0.69	3.26
Si@SnO_2_-NW^[110]^	0.32	1.698	714	1.63	–0.84	3.31
Zn@SnO_2_-NW^[110]^	6.09	1.933	224	1.45	–0.69	3.32
Pristine SnO_2_-NW^[110]^		1.970	430	1.96	–0.94	3.12
Bulk SnO_2_ (rutile)		2.053	210	2.14	–1.07	3.91
SnO_2_ (001)		1.935	556	1.81	–0.89	3.46
SnO_2_ (110)		1.964	432	1.94	–0.93	3.22

## Data Availability

Data will be made available on request.
